# Association of HLA Class I Genotypes With Severity of Coronavirus Disease-19

**DOI:** 10.3389/fimmu.2021.641900

**Published:** 2021-02-23

**Authors:** Maxim Shkurnikov, Stepan Nersisyan, Tatjana Jankevic, Alexei Galatenko, Ivan Gordeev, Valery Vechorko, Alexander Tonevitsky

**Affiliations:** ^1^Faculty of Biology and Biotechnology, HSE University, Moscow, Russia; ^2^Center for Precision Genome Editing and Genetic Technologies for Biomedicine, Pirogov Russian National Research Medical University, Moscow, Russia; ^3^Faculty of Mechanics and Mathematics, Lomonosov Moscow State University, Moscow, Russia; ^4^O.M. Filatov City Clinical Hospital, Moscow, Russia; ^5^Shemyakin-Ovchinnikov Institute of Bioorganic Chemistry, Russian Academy of Sciences, Moscow, Russia

**Keywords:** HLA class I, MHC class I, COVID-19, SARS-CoV-2, peptide presentation

## Abstract

Human leukocyte antigen (HLA) class I molecules play a crucial role in the development of a specific immune response to viral infections by presenting viral peptides at the cell surface where they will be further recognized by T cells. In the present manuscript, we explored whether HLA class I genotypes can be associated with the critical course of Coronavirus Disease-19 by searching possible connections between genotypes of deceased patients and their age at death. HLA-A, HLA-B, and HLA-C genotypes of *n* = 111 deceased patients with COVID-19 (Moscow, Russia) and *n* = 428 volunteers were identified with next-generation sequencing. Deceased patients were split into two groups according to age at the time of death: *n* = 26 adult patients aged below 60 and *n* = 85 elderly patients over 60. With the use of HLA class I genotypes, we developed a risk score (RS) which was associated with the ability to present severe acute respiratory syndrome coronavirus 2 (SARS-CoV-2) peptides by the HLA class I molecule set of an individual. The resulting RS was significantly higher in the group of deceased adults compared to elderly adults [*p* = 0.00348, area under the receiver operating characteristic curve (*AUC ROC* = 0.68)]. In particular, presence of HLA-A^*^01:01 allele was associated with high risk, while HLA-A^*^02:01 and HLA-A^*^03:01 mainly contributed to low risk. The analysis of patients with homozygosity strongly highlighted these results: homozygosity by HLA-A^*^01:01 accompanied early deaths, while only one HLA-A^*^02:01 homozygote died before 60 years of age. Application of the constructed RS model to an independent Spanish patients cohort (*n* = 45) revealed that the score was also associated with the severity of the disease. The obtained results suggest the important role of HLA class I peptide presentation in the development of a specific immune response to COVID-19.

## 1. Introduction

Human leukocyte antigen (HLA) class I molecules are one of the key mediators of the first links in the development of a specific immune response to Coronavirus Disease-19 (COVID-19) infection. Right after entering the cell, severe acute respiratory syndrome coronavirus 2 (SARS-CoV-2) induces the translation of its proteins. Some of these proteins enter the proteasomes of the infected cell, become cleaved to peptides the length of 8–12 amino acid residues, and bind to HLA class I receptors. After binding, the complex consisting of the HLA class I molecule and the peptide is transferred to the surface of the infected cell, where it can interact with the T cell receptor of CD8^+^ T lymphocytes. In response to the interaction, the CD8^+^ T lymphocyte activates and starts to divide; in 5–7 days, a population of virus-specific cytotoxic CD8^+^ T lymphocytes capable of destroying infected cells using perforins and serine proteases is formed (1). The crucial role of long-term CD8^+^ T cells activation in the immune response to COVID-19 has been recently studied in a cohort of patients who had mild disease (2, 3).

There are three main types of HLA class I receptors: HLA-A, HLA-B, and HLA-C. Receptors of every type are present in two variants inherited from parents. There exist dozens of variants of each allele of HLA-I receptors; every allele has an individual ability to recognize various foreign proteins. The distribution of alleles is population/country specific (4).

Individual combinations of HLA class I receptors essentially affect the severity of multiple infectious diseases, including malaria (5), tuberculosis (6), HIV (7), and viral hepatitis (4). There are a number of reported interconnections between the HLA genotype and the sensitivity to SARS-CoV-2. For example, the alleles HLA-B^*^07:03 (8), HLA-B^*^46:01 (9), and HLA-C^*^08:01 (10) are factors of predisposition to a severe form of the disease, and the allele HLA-C^*^15:02 is associated with a mild form (11).

Information on the interconnection of HLA class I genotype and severity of the course of COVID-19 caused by SARS-CoV-2 is sparse. A sample of 45 patients with varying severity of COVID-19 was used to confirm the results of the theoretical modeling of interaction of SARS-CoV-2 peptides with various HLA-I alleles (12). It was demonstrated that the number of peptides with a high interaction constant are connected with individual HLA genotypes: the more viral peptides with high affinity bind to HLA class I, the easier the course of the disease. It was also shown that the frequency of the occurrence of HLA-A^*^01:01 and HLA-A^*^02:01 alleles is related to the number of infections and mortality rate in different regions of Italy (13).

In the present study, we explored whether HLA class I genotypes can be a factor contributing to the critical course of COVID-19. For that, we performed HLA genotyping for *n* = 111 deceased patients with COVID-19 and the control group (*n* = 428), and searched for putative associations between genotypes and age at death. Since the total number of distinct HLA class I genotypes is too high to perform frequency-based analysis, we assigned scores to each allele based on capability of presenting SARS-CoV-2 peptides. The obtained scores allowed us to make a valid statistical comparison between HLA genotypes in groups of deceased adults (completed age at death not >60 years, *n* = 26), elderly adults (age at death over 60, *n* = 85), and the control. Special attention was paid to “extreme” cases formed by homozygous individuals by some HLA genes. Additionally, we assessed the contribution of each viral protein to the constructed risk model.

## 2. Materials and Methods

### 2.1. Design and Participants

There were 111 patients infected with COVID-19 enrolled in O.M. Filatov City Clinical Hospital, (Moscow, Russia) who died between May and July 2020. All patients had at least one positive test result for SARS-CoV-2 by reverse transcription PCR (RT-qPCR) from nasopharyngeal swabs or bronchoalveolar lavage. Patients with pathologies that led to greater morbidity or who had additional immunosuppression (patients with HIV, active cancer in treatment with chemotherapy, immunodeficiency, autoimmune diseases with immunosuppressants, and transplants) were not included in the study. Blood (2 ml) was collected by the medical practitioner from the right ventricle in an ethylenediaminetetraacetic acid (EDTA) vial *post-mortem*. Patients were divided into two groups according to their age at death: adults (age ≤ 60, *n* = 26) and elderly adults (age >60, *n* = 85).

The control group of 428 volunteers was established with the use of electronic HLA genotype records of the Federal Register of Bone Marrow Donors (Pirogov Russian National Research Medical University). All patients or their next of kin gave informed consent for participation in the study.

The study protocol was reviewed and approved by the Local Ethics Committee at the Pirogov Russian National Research Medical University (Meeting No. 194 of March 16 2020, Protocol No. 2020/07); the study was conducted in accordance with the Declaration of Helsinki.

### 2.2. Human Leukocyte Antigen Class I Genotyping With Targeted Next-Generation Sequencing

Genomic DNA was isolated from frozen collected anticoagulated whole blood samples using the QIAamp DNA Blood Mini Kit on the automatic workstation QIACube (QIAGEN GmbH, Hilden, Germany). HLA-A, HLA-B, and HLA-C genes were sequenced with the MiSeq platform (Illumina, San Diego, CA, USA) through exons 2–4 in both directions using reagent kit HLA-Expert (DNA-Technology LLC, Moscow, Russia) and annotated using the database of the human major histocompatibility complex (MHC) sequences IMGT/HLA v3.41.0 (14). Processed genotype data are available in Supplementary Table 1.

### 2.3. Severe Acute Respiratory Syndrome Coronavirus 2 Protein Sequences

Publicly available SARS-CoV-2 proteomes derived from patients infected in Moscow (*n* = 79) were obtained from GISAID (15) (full list of IDs is presented within Supplementary Table 2). Clustal Omega v1.2.4 was used to construct multiple sequence alignment for each viral protein (16). The obtained alignment had no gaps and rare mutations: only 117 out of 9,719 positions (1.2%) contained more than one amino acid variant. Moreover, distribution of non-major amino acid fractions at mutation sites was also concentrated near zero: maximum fraction was equal to 22.8% (18 out of 79 viruses), 0.95 quantile was equal to 5.1% (four viruses) and upper quartile was equal to 1.3% (one virus with mismatched amino acids).

Since many research groups use the reference SARS-CoV-2 genome and proteome sequences (Wuhan-Hu-1, NCBI Reference Sequence: NC_045512.2), we compared the obtained alignment with the mentioned reference. Two protein sequences differed in four positions (0.04%) located within the NSP12, N, and S viral proteins. Such differences can indeed lead to functional diversity of analyzed proteins, but will be totally negligible for further analysis.

### 2.4. Prediction of Viral Peptides and Assessment of Their Binding Affinities to HLA Class I Molecules

We applied the procedure described by Nguen et al. (17) to the consensus protein sequences of viruses isolated from patients in Moscow. Specifically, for each amino acid of each viral protein, we assessed the probability of proteasomal cleavage in the considered site using NetChop v3.1 (18). The set of viral peptides was generated by taking all possible 8- to 12-mers with proteasomal cleavage probability <0.1 at both ends of a sequence.

Binding affinities were predicted using netMHCpan v4.1 (19) for all viral peptides (*n* = 15,314) and HLA alleles present in our cohorts of deceased and control patients (*n* = 107). Peptides with a weak binding affinity to all considered alleles were discarded (*IC*_50_ affinity values above 500 nM as recommended by netMHCpan developers). For the remaining 6,548 peptides, all affinities were inverted, multiplied by 500 and log_10_-transformed. Thus, the resulting score was equal to zero for peptides with a weak binding affinity threshold (500 nM) and equal to one for a high binding affinity (50 nM). Raw and processed matrices are presented in Supplementary Table 3.

### 2.5. Statistical Analysis

Allele frequencies in considered cohorts were estimated by dividing the number of occurrences of a given allele in individuals by the doubled total number of individuals (i.e., identical alleles of homozygous individuals were counted as two occurrences). The following functions from the scipy.stats Python module (20) were used to conduct statistical testing: fisher_exact for Fisher's exact test, mannwhitneyu for Mann-Whitney *U* test. The Benjamini-Hochberg procedure was used to perform multiple testing corrections. Principal component analysis was conducted with the scikit-learn Python module (21). Permutation test for assessing significance of area under the receiver operating characteristic curve (AUC ROC) values was done with *n* = 10^6^ label permutations. Plots were constructed with Seaborn and Matplotlib (22).

## 3. Results

### 3.1. Distribution of HLA Class I Gene Alleles in the Cohort of Deceased COVID-19 Patients and the Control Group

We performed HLA class I genotyping for *n* = 111 deceased patients with confirmed COVID-19 (Moscow, Russia) and the control group consisting of volunteers (*n* = 428). Deceased patients were divided into two groups: adults (age at death ≤ 60 years) and elderly adults (age at death over 60 years). Demographic and clinical data of these cohorts are summarized in Table 1. Although patients with severe comorbidities were excluded from the study, 76.6% of deceased patients had at least one underlying disease. Only cerebrovascular disease had a statistically significant odds ratio when comparing groups of adults and elderly adults (3.8 vs. 34.1%, Fisher's exact test *p* = 1.89 × 10^−3^). Other cardiovascular diseases like coronary artery disease and heart failure were also more frequent in the group of elderly individuals which, however, was not statistically significant. Interestingly, arterial hypertension was diagnosed in 11.5% of adult patients and 24.7% of older adults, which was generally less than populational level in Russia (about 50%) (23). Percentage of diabetes cases was about 3.5% in both analyzed groups, which is a typical value for the current population of Russia (24). Also, frequencies of chronic kidney disease (stages 4–5) in both groups (23.1% for adults and 16.5% for elderly individuals) was significantly higher compared to the background populational value (about 0.05%) (25).

**Table 1:** Demographic and clinical data in the cohort of deceased patients with COVID-19.

	**Deceased, age ≤ 60, *n* = 26**	**Deceased, age > 60, *n* = 85**	***p***
Age, completed years, median (Q25–Q75)	54.5 (45.25–57)	79 (70–84)	
**Gender**			
Female	10 (38.5%)	42 (49.4%)	0.375
Male	16 (61.5%)	43 (50.6%)	0.375
**Comorbidities**			
None	8 (30.8%)	18 (21.2%)	0.305
1	11 (42.3%)	43 (50.6%)	0.507
>1	7 (26.9%)	24 (28.2%)	1.000
**Cardiovascular disease**			
Arterial hypertension	3 (11.5%)	21 (24.7%)	0.184
Coronary artery disease	1 (3.8%)	9 (10.6%)	0.448
Cardiac infarction	1 (3.8%)	2 (2.4%)	0.555
Heart failure	0 (0.0%)	9 (10.6%)	0.113
Stroke	2 (7.7%)	7 (8.2%)	1.000
Cerebrovascular disease	1 (3.8%)	29 (34.1%)	**1.89 × 10^−3^**
**Metabolic disease**			
Obesity	1 (3.8%)	1 (1.2%)	0.415
Diabetes	1 (3.8%)	3 (3.5%)	1.000
Chronic obstructive pulmonary disease	2 (7.7%)	3 (3.5%)	0.333
**Neoplasma**	5 (19.2%)	8 (9.4%)	0.179
**Others**			
Alcoholic liver disease	2 (7.7%)	2 (2.4%)	0.233
Gastric ulcer	3 (11.5%)	3 (3.5%)	0.140
Chronic kidney disease, stages 4–5	6 (23.1%)	14 (16.5%)	0.560

*Comorbidity ratios were compared with Fisher's exact test. Bold values indicate p < 0.05*.

**Figure 1:**
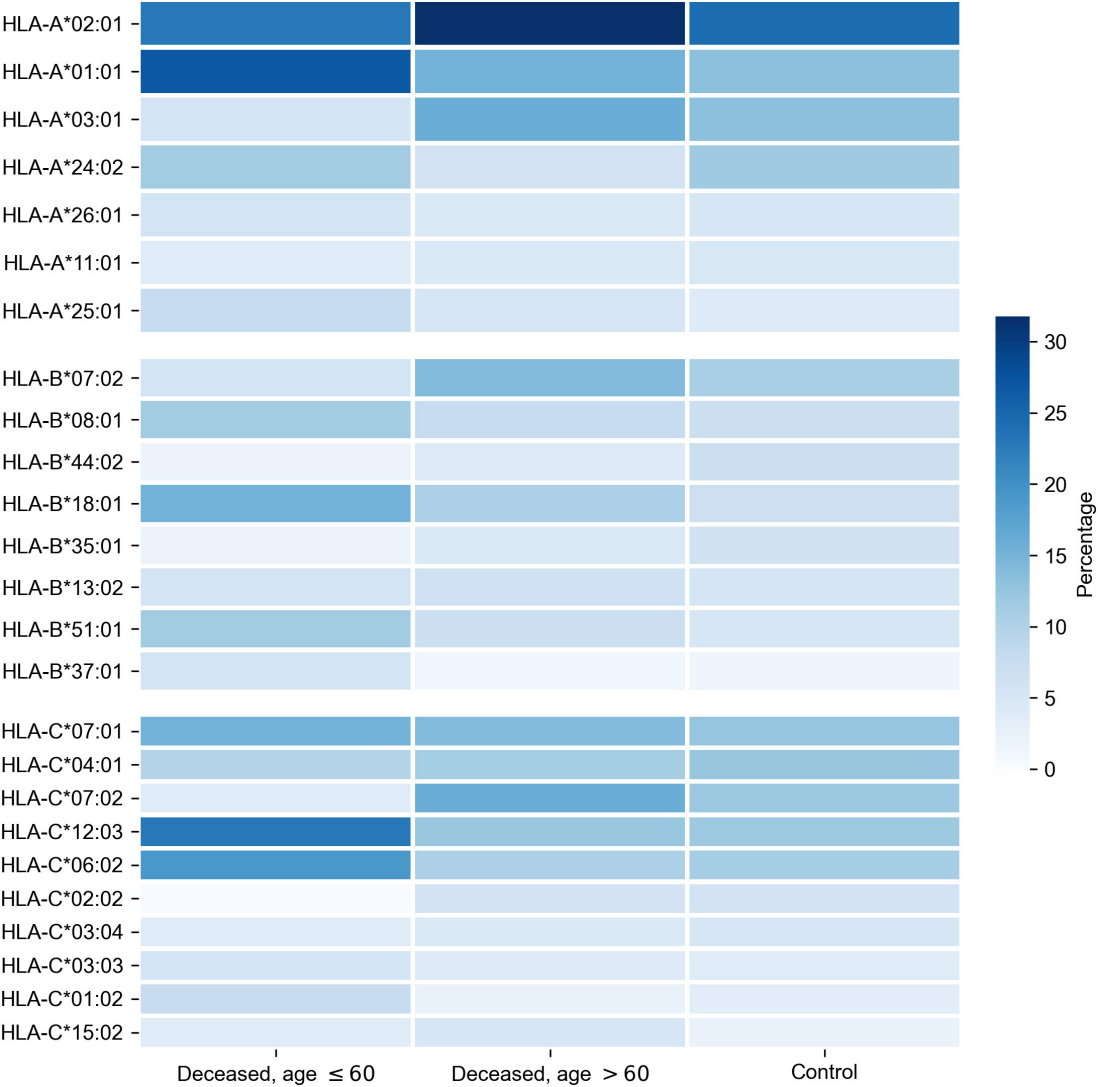
Distribution of HLA-A, HLA-B, HLA-C alleles in cohorts of deceased COVID-19 patients and the control group. Alleles with frequency over 5% in at least one of three considered groups are presented.

First, we tested whether frequency of a single allele can differentiate individuals from three groups: adult patients who died from COVID-19, elderly patients who died from COVID-19, and the control group. Distribution of major HLA-A, HLA-B, and HLA-C alleles in these three groups is summarized in Figure 1. Fisher's exact test was used to make formal statistical comparisons. As a result, we found that for all possible group comparisons not a single allele had an odds ratio, which can be considered statistically significant after multiple testing correction (all corrected *p*-values were equal to 1). However, few of them were differentially enriched if no multiple testing corrections were applied (Supplementary Table 4).

### 3.2. Binding Affinities of Viral Peptides to HLA Class I Molecules

Since sizes of considered cohorts were insufficient for performing frequency analysis at the level of full HLA class I genotypes, we transformed patient genotypes from discrete space to numerical units associated with the potential of interactions with SARS-CoV-2 peptides. To implement this idea, we first constructed a matrix of binding affinities of viral peptides to HLA-A, HLA-B, and HLA-C alleles. For that, we first made computational predictions of viral peptides derived from SARS-CoV-2 strains isolated in Moscow. Then, binding affinities were calculated for each of the predicted peptides and each allele present in patients from the analyzed cohorts.

**Figure 2:**
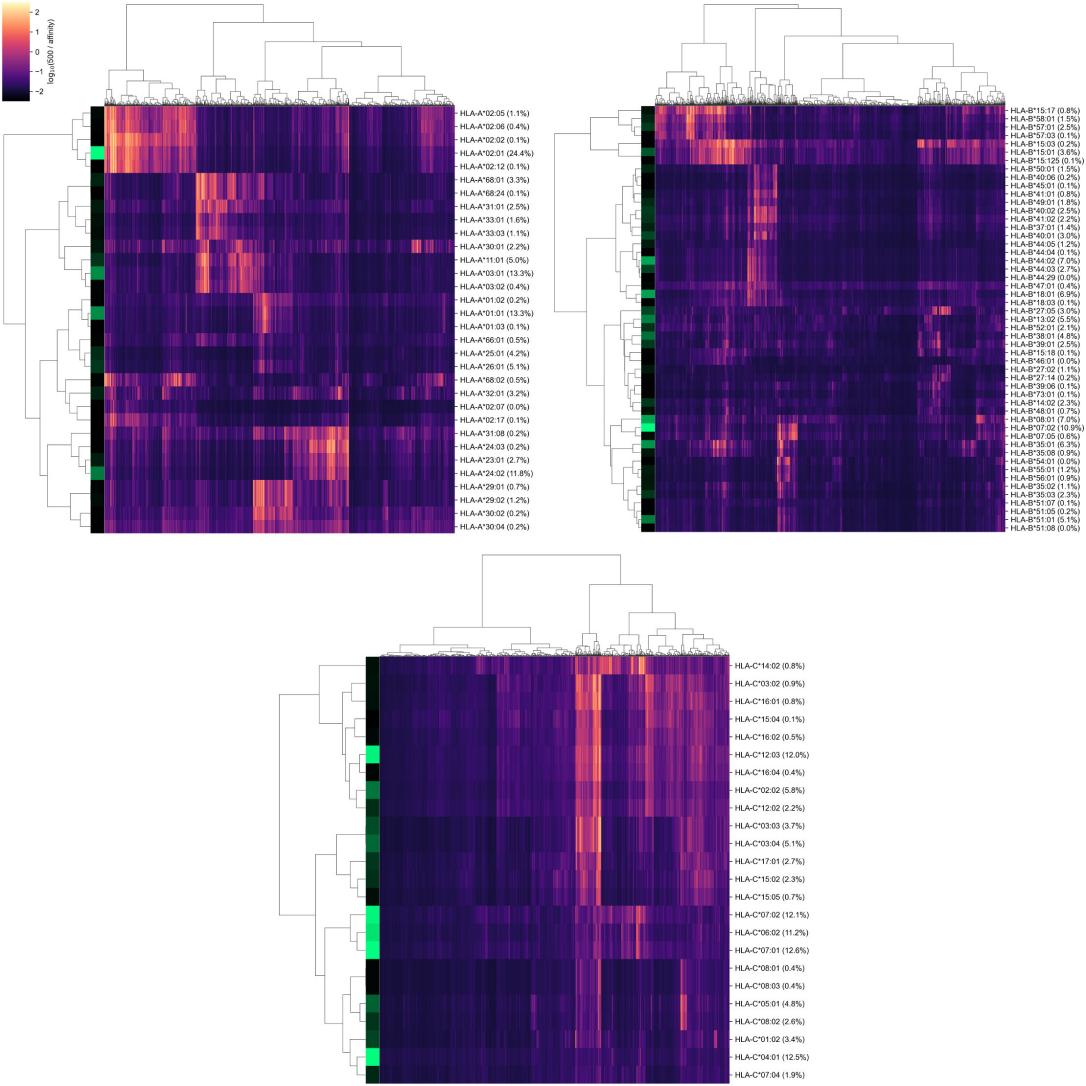
Hierarchical clustering of HLA-A, HLA-B, HLA-C gene alleles and SARS-CoV-2 peptides according to binding affinity matrix. Shades of green in vertical stripes and percents in brackets represent frequency of an allele in the control group. Zero percents refer to rare alleles found only in the group of deceased patients.

As a result, we obtained a matrix containing affinity values for 6,548 peptides and 107 alleles of genes from major HLA class I. To establish a positive relationship between values from the matrix and binding potential, all affinities were inverted and scaled by a value of 500 nM (the conventional threshold for binding ability). Simultaneous hierarchical clustering was applied to identify groups of similar peptides and HLA-A, HLA-B, and HLA-C alleles (Figure 2). As can be seen, both alleles and peptides clearly formed several dense clusters. The most presented alleles of HLA-A (HLA-A^*^01:01, HLA-A^*^02:01, HLA-A^*^03:01, and HLA-A^*^24:02) fell in different clusters, while for HLA-B and HLA-C, some major alleles were grouped together (e.g., HLA-B^*^07:02, HLA-B^*^08:01, HLA-C^*^06:02, HLA-C^*^07:01, and HLA-C^*^07:02).

**Figure 3:**
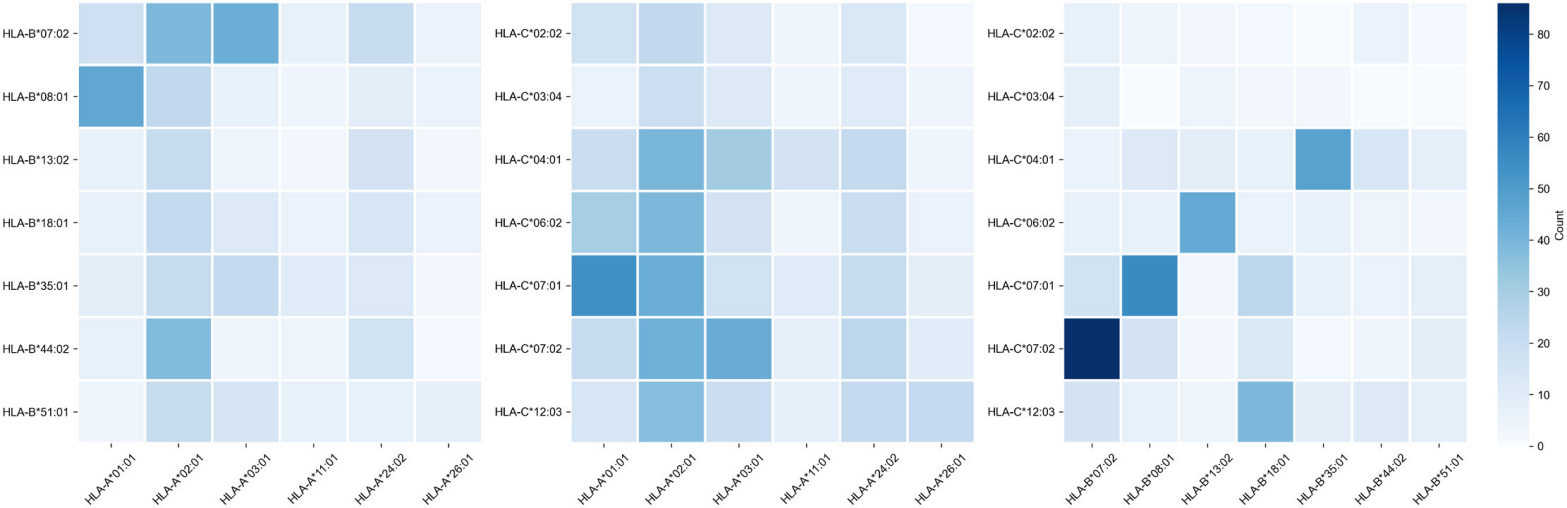
Contingency table of allele counts in the control group. Alleles with frequency over 5% in the control group are presented.

Note that alleles with similar peptide binding profiles can be linked to different alleles of remaining genes. For example, consider closely clustered alleles HLA-C^*^06:02, HLA-C^*^07:01, and HLA-C^*^07:02. From the analysis of the contingency table of allele pairs in the control group (Figure 3), it follows that each of these alleles has its own spectrum of associated alleles. Specifically, HLA-C^*^06:02 usually appears with HLA-B^*^13:02 (Fisher's exact test *p* = 3.18 × 10^−14^), HLA-C^*^07:01 is linked to HLA-A^*^01:01 (*p* = 2.97 × 10^−7^) and HLA-B^*^08:01 (*p* = 3.15 × 10^−14^), while HLA-C^*^07:02 is coupled with HLA-A^*^03:01 (*p* = 9.66 × 10^−4^) and HLA-B^*^07:02 (*p* = 2.72 × 10^−26^). Interestingly, such linked alleles can have different peptide binding patterns (e.g., see weakly overlapped bars for HLA-A^*^01:01 and HLA-A^*^03:01 in Figure 2).

### 3.3. Risk Score Based on Peptide-HLA Binding Affinity Is Associated With Early COVID-19 Deaths

For each of the considered HLA-A, HLA-B, and HLA-C alleles, we obtained the list of binding affinities to 6,548 unique SARS-CoV-2 peptides. In order to calculate aggregate information on the potential of presenting SARS-CoV-2 peptides by each allele, we used principal component analysis (PCA). In this framework 6,548-element affinity vectors are replaced by the most informative linear combinations of their components. For HLA-A and HLA-C, we found four principal components (PCs), each of which explained at least 5% of data variance, while for HLA-B, the number of essential components was equal to five (Supplementary Table 5). Signs of components were set in the way to achieve positive correlation of component values with age of death of deceased patients.

**Figure 4:**
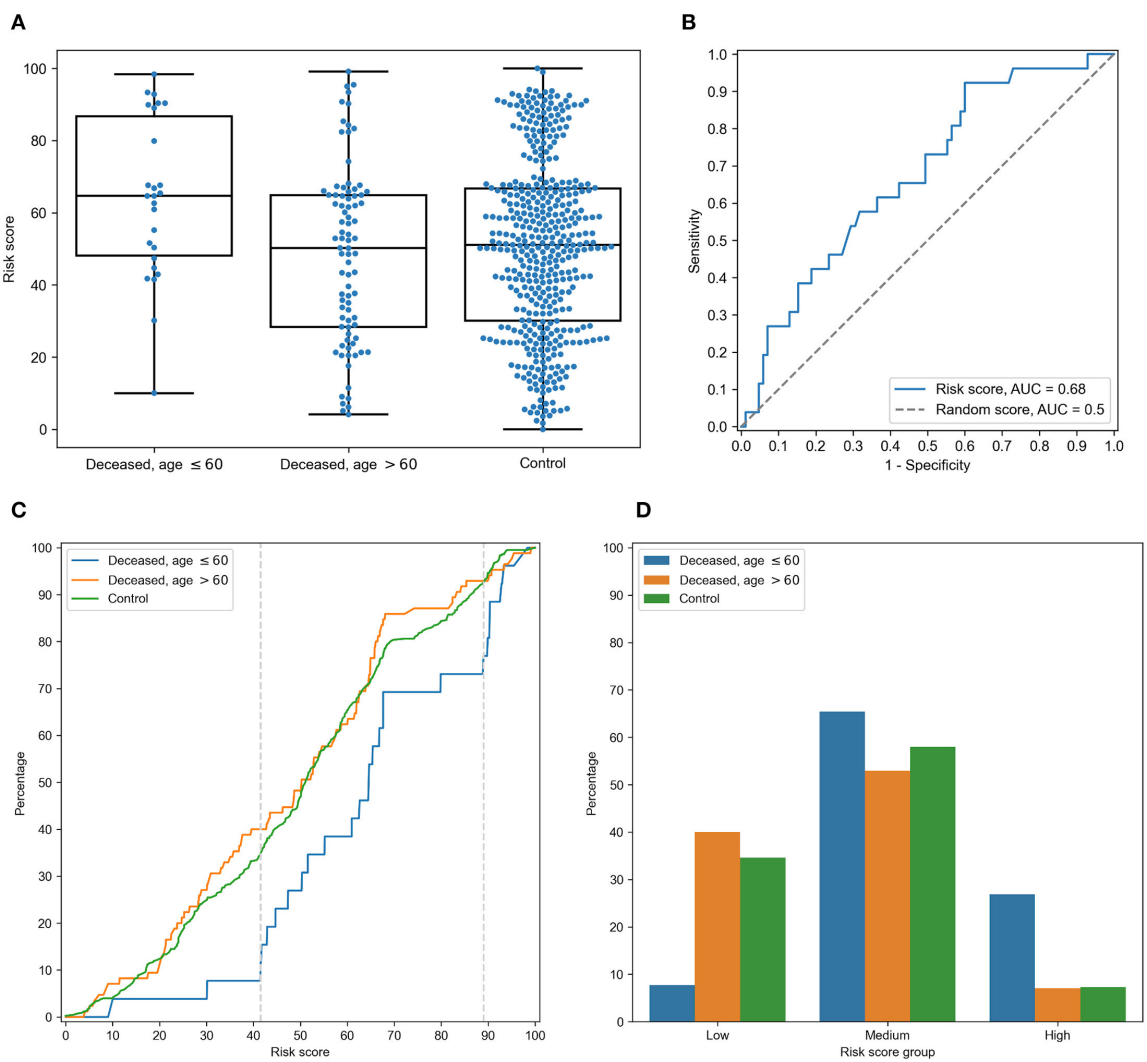
Risk score (RS) separates groups of adult and elderly patients. **(A)** Distribution of RS in adult, elderly and control patient groups. **(B)** Receiver operating characteristic curve for RS separating patients from adult and elderly groups. **(C)** Empirical distribution function of RS in three patient groups. Vertical dotted lines at RS = 41 and RS = 89 define ranges for three RS groups. **(D)** Distribution of three patient groups over low, medium and high RS.

For each individual, HLA class I gene, and PC, we summed PC values associated with two corresponding alleles. After that, we analyzed differences of obtained scores in adult and elderly patients who died from COVID-19. Three of the resulting PCs demonstrated statistically significant differences according to the Mann-Whitney *U* test. This list included the second and third PCs of HLA-A, and the fourth PC of HLA-C, while no PCs of HLA-B separated the analyzed groups significantly. As an aggregate risk score (RS), we considered the sum of these three components (for convenience, we linearly scaled the range of RS to the [0, 100] interval). The obtained score also significantly separated groups with *p* = 3.48 × 10^−3^ (U test) and AUC ROC equal to 0.68 (permutation test *p* = 3.10 × 10^−3^), see Figures 4A,B (full information is listed in Supplementary Table 6). Interestingly, the difference of RS distributions in the cohort of adult patients and the control group was also statistically significant (*U* test *p* = 3.31 × 10^−3^), while the difference between the elderly and control groups was not (*p* = 0.283).

In order to characterize the association between RS and age at death more precisely, we partitioned the range of RS into three groups: low, medium, and high (Figures 4C,D). The lower and higher thresholds were calculated in a way to minimize *p*-value for Fisher's exact test applied to the number of adult and elderly patients in the whole cohort and in the low/high risk groups, respectively. Interestingly, such partitioning led to significant separation of adult patients both from the elderly and control subjects in the low and high risk groups, while no significance was found within the middle group (Supplementary Table 7).

Then, we performed enrichment analysis to identify alleles significantly contributing to each of the RS groups (Table 2). As it can be seen, frequencies of several alleles were dramatically higher in some RS groups. Specifically, HLA-A^*^02:01 and HLA-A^*^03:01 were highly overrepresented in the low risk group and completely absent in the high risk group, while the most enriched allele in the high risk group was HLA-A^*^01:01. Reciprocally to HLA-A^*^02:01 and HLA-A^*^03:01 cases, not a single individual in the low risk group carried the HLA-A^*^01:01 allele. Complete information on allele frequencies in the RS groups is presented in Supplementary Figure 1.

**Table 2:** Enrichment analysis of risk score groups.

		**Deceased**			**Control**		
**RS group**	**Allele**	**Percentage, RS group (%)**	**Percentage, cohort (%)**	***p***	**Percentage, RS group (%)**	**Percentage, cohort (%)**	***p***
Low	HLA-A^*^03:01	27.0	13.5	**7.54 × 10^−3^**	24.2	13.3	**1.60 × 10^−5^**
	HLA-A^*^02:01	44.6	29.7	**0.0146**	37.9	24.4	**7.65 × 10^−6^**
	HLA-A^*^11:01	10.8	4.5	0.051	10.7	5.0	**7.59 × 10^−4^**
	HLA-B^*^15:01	5.4	3.6	0.350	6.7	3.6	**0.022**
	HLA-C^*^08:02	1.4	0.9	0.580	5.0	2.6	**0.0334**
	HLA-C^*^05:01	1.4	2.7	0.870	8.1	4.8	**0.0230**
Medium	HLA-A^*^01:01	21.8	18.0	0.239	19.2	13.3	**2.65 × 10^−3^**
	HLA-A^*^24:02	8.9	7.2	0.360	17.0	11.8	**5.06 × 10^−3^**
High	HLA-A^*^01:01	54.2	18.0	**2.15 × 10^−4^**	29.0	13.3	**1.50 × 10^−3^**
	HLA-B^*^37:01	12.5	2.3	**0.0331**	1.6	1.4	0.600
	HLA-C^*^06:02	29.2	12.6	**0.0364**	11.3	11.2	0.557
	HLA-A^*^24:02	20.8	7.2	**0.0400**	25.8	11.8	**2.88 × 10^−3^**
	HLA-A^*^26:01	16.7	5.0	**0.0459**	25.8	5.1	**4.23 × 10^−7^**
	HLA-B^*^08:01	20.8	8.6	0.0680	16.1	7.0	**0.0148**
	HLA-B^*^38:01	12.5	3.6	0.0780	16.1	4.8	**1.35 × 10^−3^**
	HLA-C^*^07:01	20.8	14.4	0.283	29.0	12.6	**8.380 × 10^−4^**
	HLA-C^*^12:03	20.8	14.9	0.304	32.3	12.0	**5.530 × 10^−5^**
	HLA-A^*^25:01	4.2	5.9	0.772	14.5	4.2	**2.079 × 10^−3^**
	HLA-B^*^18:01	8.3	11.7	0.790	16.1	6.9	**0.0134**

*One-sided Fisher's exact test was used to identify HLA-A, HLA-B, and HLA-C alleles overrepresented in each of the RS groups in cohorts of deceased and control individuals. Data shown for alleles with p < 0.05 in at least one of cohorts and with frequency over 5% in at least one of four considered conditions. Bold values indicate p < 0.05*.

Finally, we assessed the contribution of individual peptides from different viral proteins to the RS. Contribution of a peptide to RS was calculated as an absolute value of the sum of corresponding PC coefficients (PC2 and PC3 for HLA-A, and PC4 for HLA-C). Then, the set of the most RS contributing peptides was composed by taking the top 5% of peptides from the corresponding distribution. The results of the procedure are summarized in Table 3: distribution of peptides with the strongest contributions over SARS-CoV-2 proteins was similar to the one calculated for all peptides after multiple testing correction. Without the correction, only non-structural protein 8 (NSP8) had a statistically significant odds ratio. Thus, considered peptides were spread over proteins without any significant dependence on contribution to the RS.

**Table 3:** Distribution of peptides and viral proteins and their contribution to the RS.

**Protein**	**Percentage, important peptides (%)**	**Percentage, all peptides (%)**	**Odds ratio**	***p***	**FDR**
NSP3	19.5	19.6	1.0	1.000	1.000
Spike	9.5	12.2	0.8	0.164	0.682
NSP12	13.7	10.4	1.4	0.0647	0.539
NSP4	5.2	7.2	0.7	0.186	0.582
NSP13	5.5	6.2	0.9	0.724	0.953
NSP2	6.1	5.3	1.2	0.528	0.825
NSP14	4.0	5.2	0.8	0.439	0.784
NSP6	3.7	4.7	0.8	0.423	0.814
NS3	2.1	3.5	0.6	0.215	0.597
NSP16	4.3	3.3	1.3	0.339	0.770
M	5.2	3.1	1.7	0.0532	0.665
N	3.7	3.0	1.2	0.508	0.847
NSP5	2.4	2.9	0.8	0.737	0.921
NSP15	4.3	2.6	1.7	0.0800	0.500
NSP8	4.3	2.0	2.2	**9.84 × 10^−3^**	0.246
NS7a	1.8	1.5	1.2	0.640	0.889
NSP1	1.5	1.4	1.1	0.806	0.959
E	0.9	1.2	0.8	1.000	1.000
NS8	0.3	1.2	0.3	0.272	0.679
NSP9	0.6	1.1	0.6	0.584	0.859
NSP7	0.0	0.8	0.0	0.178	0.637
NS6	0.3	0.6	0.5	1.000	1.000
NS7b	0.0	0.5	0.0	0.405	0.845
NSP10	1.2	0.5	2.6	0.083	0.417
NSP11	0.0	0.0	0.0	1.000	1.000

*Fisher's exact test was used to compare fractions of each protein in the most RS contributing and all peptides. Bold values indicate p < 0.05*.

### 3.4. The RS Is Associated With Severity of COVID-19 in the Cohort of Spanish Patients

**Figure 5:**
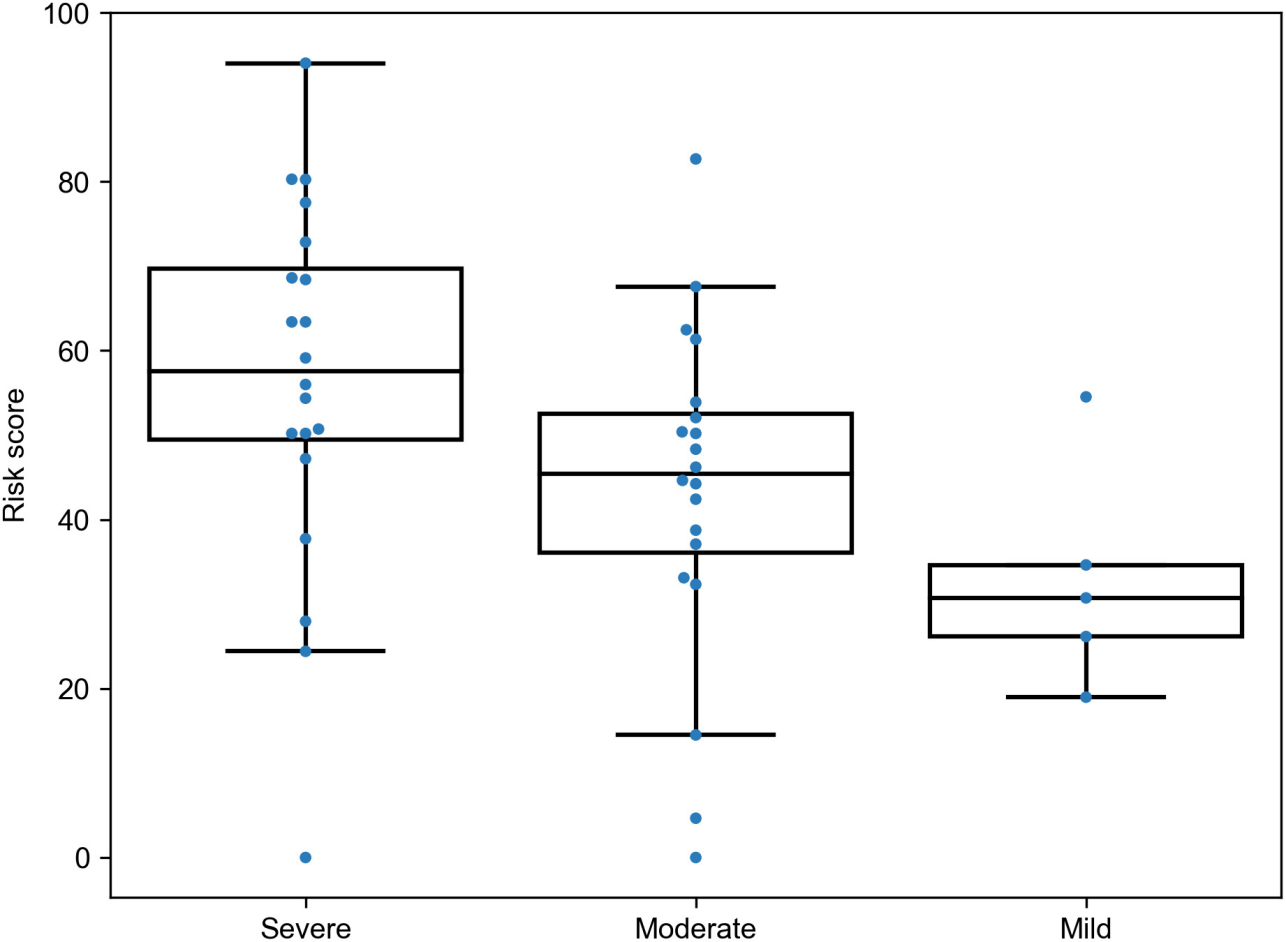
Risk score (RS) in groups of severe, moderate, and mild COVID-19 patients from the Spanish cohort.

To see whether the proposed RS was associated with different patterns of the disease severity, we re-analyzed data from a recently published study on the role of HLA class I genotypes in COVID-19 in a cohort of Spanish patients (12). The data included genotypes of patients with severe (*n* = 20), moderate (*n* = 20), and mild (*n* = 5) SARS-CoV-2 infection. The RS model was applied to the data with no re-tuning of coefficients, and the same PCA weights were used for cohort-specific alleles. As a results, we found statistically significant dominance of RSs in patients with severe symptoms compared to moderate (*U* test *p* = 0.0157) and mild (*p* = 0.0161) patients, while, despite the matching direction, no statistical significance was found for the comparison of patients with moderate and mild infection (*p* = 0.0926), possibly due to the low sample size (Figure 5). Thus, the developed model allowed us to find dependencies between HLA class I genotypes and severity of the disease in the independent patient cohort from another population.

### 3.5. Human Leukocyte Antigen Class I Homozygosity Is a Double-Edged Sword for COVID-19 Risk

When analyzing the high RS group, we noticed that more than half (five out of eight) deceased patients containing HLA-A^*^01:01 alleles were homozygous by this allele, while the medium group had not a single individual homozygous by HLA-A^*^01:01 (Fisher's exact test *p* = 0.0103). The distribution of homozygous individual by HLA-A^*^01:01 among the groups of deceased patients and the control group proved its negative role. It turned out that the distribution in the deceased group (4 out of 26 patients who died <60, and 1 out of 85 patients who died >60) leads to two statistically significant differences: *p*-value of Fisher's exact test comparing the adults group with the elderly group equals 3.10 × 10^−3^, and *p*-value of the test comparing the adults group and the control group equals 0.0104 (8 out of 428 members of the control group were homozygous by HLA-A^*^01:01). However, the difference between the elderly group and the control group is statistically insignificant (*p* = 0.155). Interestingly, there were no other statistically significant differences in the distribution of homozygosity between the groups.

Generally, the average age of death for patients homozygous by any allele was significantly less compared to heterozygous ones (Mann-Whitney *U* test *p* = 6.45 × 10^−3^, Supplementary Figure 2). Also, the fraction of homozygous patients was higher in the group of deceased adults (42.3%) compared both to elderly patients (15.3%, Fisher's exact test *p* = 6.03 × 10^−3^) and the control group (19.2%, *p* = 9.80 × 10^−3^). Difference between the elderly and control groups was not statistically significant (*p* = 0.448).

However, the low risk group also contained homozygous individuals: all homozygosity cases by HLA-A^*^02:01 (six cases) and HLA-A^*^03:01 (two cases) alleles were associated with low risk. These assignments were in agreement with age of death: only one patient homozygous by HLA-A^*^02:01 had not passed the 60 years age of death threshold. Thus, homozygosity by HLA class I genes is generally associated with poor prognosis except for some alleles like HLA-A^*^02:01 and HLA-A^*^03:01 with “relevant” peptide-binding profiles.

## 4. Discussion

In the current study, we presented the characteristics of a large cohort of deceased patients with COVID-19 from O.M. Filatov City Clinical Hospital in Moscow, Russia. The clinical characteristics of these patients indicated that the HLA genotype, age, and underlying diseases were the most important risk factors for death. In our population, the median age of deceased patients was 73.0 years. Three previous studies reported an average age in non-survivors to be 78.0, 65.8, and 70.7 years old, respectively (26–28). Our data are in line with the literature reaffirming that advanced age is one of the strongest predictors of death in patients with SARS-CoV-2 (29).

The majority of deceased patients at age <60 were men (61.5%), while the populational level for this age category in Russia was 48% (30). Such imbalance is in agreement with information that COVID-19 is more prevalent in men (29). This trend continued in the group of the elderly adults where sex distribution was close to uniform, while only 37.5% of the population from this age group was male.

Only 18 deceased elderly patients (21.2%) had no comorbidities. A number of previous studies mentioned a high percentage of comorbidities in a group of patients with the severe course of COVID-19 (26, 30, 31). At the same time, we were unable to find statistically significant differences in fractions on different comorbidities between the adult and elderly groups except the percentage of cerebrovascular disease. Specifically, only one adult (3.8%) had suffered from this disease compared to 29 individuals (34.1%) from the elderly group. The results of previously conducted meta-analysis of 1,558 individuals infected by COVID-19 already highlighted cerebrovascular disease as a risk factor for COVID-19 infection; however, this was not associated with increased mortality (32). It is well-known that frequency of cardiovascular comorbidities increases with age (33), and in total with age-related decrease in T cell receptor repertoires, it negatively affects the prognosis of COVID-19 (34).

Differences of frequencies of HLA class I alleles were not statistically significant after multiple testing corrections both in comparisons between deceased patients and control groups, and the deaths of adults vs. elderly subjects. Previously, Wang with co-authors performed comparisons of allele frequencies between groups of Chinese individuals infected with COVID-19 and controls, which resulted in significant difference only for rare alleles, such as HLA-C^*^07:29 and HLA-B^*^15:27 (35). Thus, the analysis of the whole HLA class I genotype should be performed to identify possible associations with clinical information.

Since the available cohort size is insufficient to deeply cover possible genotypes (two alleles for each of HLA-A, HLA-B, and HLA-C genes), we assigned a numerical value to each allele associated with the aggregate binding affinity of viral peptides to the corresponding receptor. The obtained RS separated adult patients who died due to COVID-19 from both the elderly subjects and the control group with a statistical significance. A conceptually similar technique was used by Iturrieta-Zuazo et al.: allele score was calculated as a number of tightly binding viral peptides (affinity <50 nM) (12). However, our PC-based approach can be more robust since it does not depend on any threshold. Application of the constructed model to their data additionally validated robustness of the approach, allowing us to discriminate groups of patients with severe, moderate, and mild disease course in a statistically significant way.

To identify extreme values of RS, we split its range into low, medium, and high risk groups. Three HLA-A alleles were highly overrepresented in these groups: HLA-A^*^02:01 and HLA-A^*^03:01 were tightly associated with low risk while HLA-A^*^01:01 contributed to the high risk group. Connection of HLA-A^*^01:01 and HLA-A^*^02:01 alleles with COVID-19 morbidity and mortality was already mentioned in the existing literature, namely, frequency of HLA-A^*^01:01 in Italian regions positively correlated both with the number of COVID-19 cases and deaths, while significant negative correlation was observed for HLA-A^*^02:01 (13).

An interesting illustration for the role of HLA-A^*^01:01 and HLA-A^*^02:01/HLA-A^*^03.01 was discovered during the analysis of homozygous individuals. Homozygosity only by HLA-A^*^01:01 as well as homozygosity by any allele were significantly associated with the earlier age of death compared to the corresponding heterozygous individuals. Such observations were already noted for some other infectious diseases. For example, a limited number of recognized peptides due to HLA class I homozygosity led to a higher progression rate from HIV to AIDS (36). On the contrary, we found that only one out of eight HLA-A^*^02:01 or HLA-A^*^03:01 individuals with homozygosity died before 60 years of age. This fact can also be observed in the dataset recently published by Warren with co-authors (37): none out of the four COVID-19 patients homozygous by HLA-A^*^02:01 or HLA-A^*^03:01 had a severe course of COVID-19 and were admitted to the intensive care unit. Moreover, the control non-infected group contained significantly a higher fraction of such homozygotes: seven out of 26 non-infected controls (26.9%) vs. four out of 100 infected (4%) patients (Fisher's exact test *p* = 1.40 × 10^−3^). Thus, presentation of “important” viral peptides with doubled intensity can enhance immune response showing that HLA class I homozygosity can act like a double-edged sword.

Since RS was constructed as a linear combination of peptide-HLA binding affinities, it was possible to rank peptides according to their contribution to the RS. Only one protein, NSP8, had a statistically significant fraction of RS contributing peptides, which, however, was negligible after multiple testing corrections. Thus, the most “important” peptides were spread across viral proteins proportional to their total fractions (see Table 3). Grifoni et al. also made a computational prediction of CD8^+^ T cell epitopes by taking the top 1% of peptides according to their binding affinity to 12 common HLA class I alleles (38). In total, 628 peptides were predicted, which significantly intersected with our list of the most “important” peptides: 198 out of 328 epitopes predicted by our analysis were present in the list provided (38). Further experimental validation with the use of “megapools” demonstrated strong T cell immune response to these peptides in a cohort of 20 recovered patients (39). As in our case, immunopeptidome was not limited to any particular protein.

## Data Availability Statement

The original contributions presented in the study are included in the article/Supplementary Material, further inquiries can be directed to the corresponding author/s.

## Ethics Statement

The studies involving human participants were reviewed and approved by the Local Ethics Committee at the Pirogov Russian National Research Medical University (Meeting No. 194 of March 16 2020, Protocol No. 2020/07). The patients/participants provided their written informed consent to participate in this study.

## Author Contributions

MS, SN, and AT: conceptualization and validation. MS, SN, TJ, AG, IG, VV, and AT: methodology, investigation, and writing (review and editing). SN and AG: software and formal analysis. MS and SN: writing (original draft). All authors contributed to the article and approved the submitted version.

## Conflict of Interest

The authors declare that the research was conducted in the absence of any commercial or financial relationships that could be construed as a potential conflict of interest.
