# Supplementary figures and images for: Association of HLA Class I Genotypes With Severity of Coronavirus Disease-19
Source: Front Immunol. 2021 Feb 23;12:641900. doi: 10.3389/fimmu.2021.641900 (PMC7959787; doi:10.3389/fimmu.2021.641900)

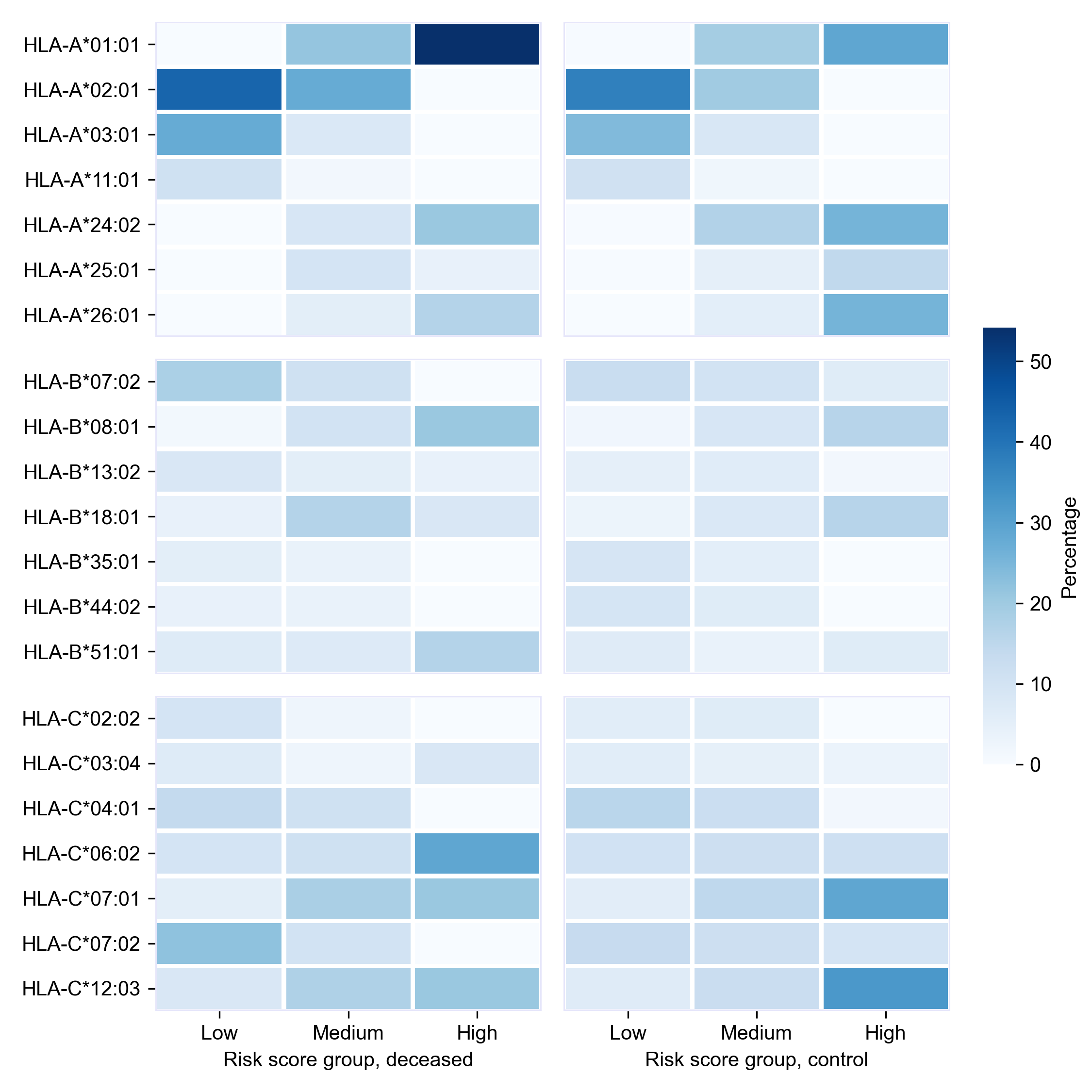

Supplement: Supplementary file 8 [file Image_1.TIF]

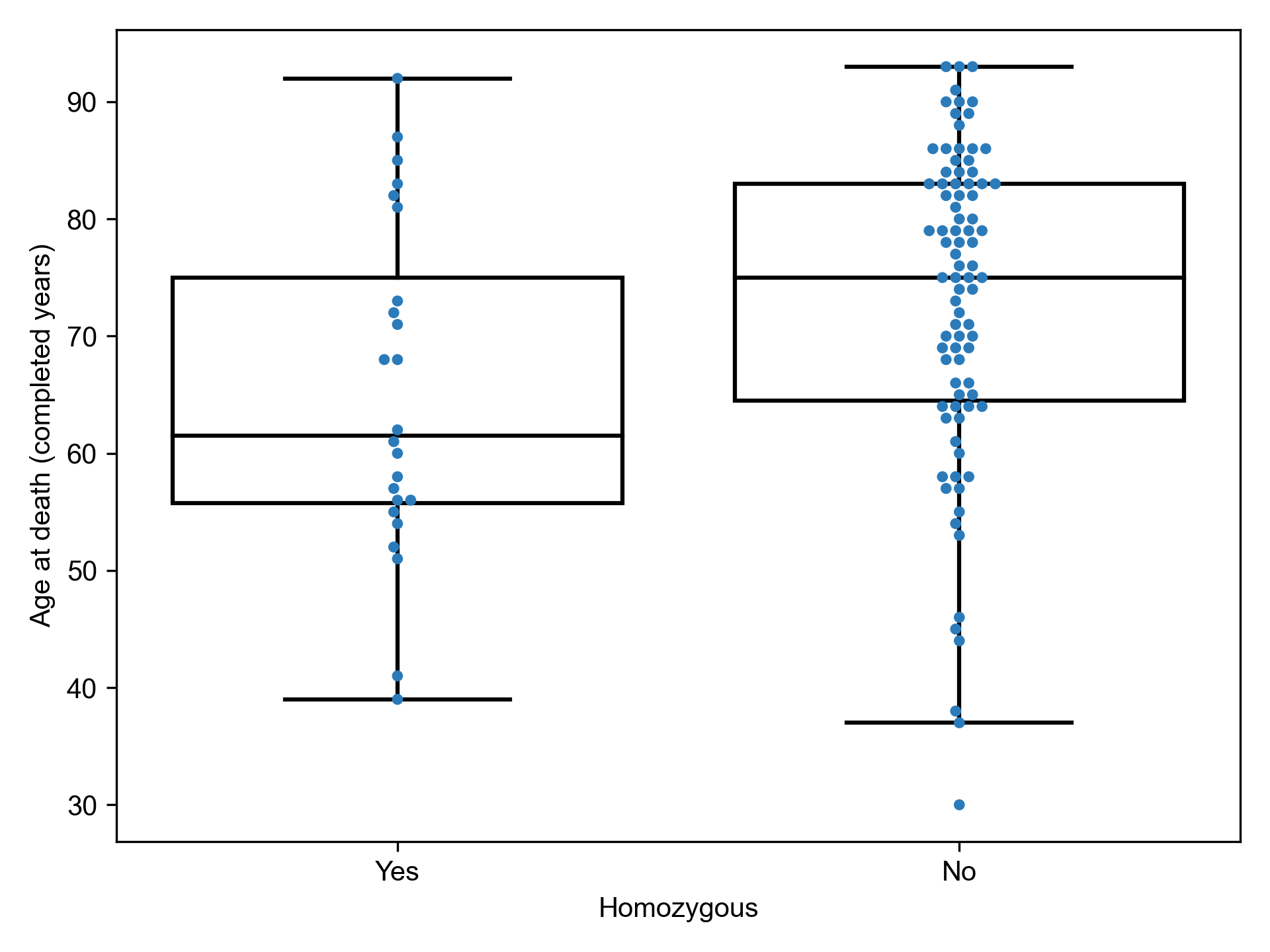

Supplement: Supplementary file 9 [file Image_2.TIF]
