# Supplementary material for: Association of HLA Class I Genotypes With Severity of Coronavirus Disease-19
Source: Front Immunol. 2021 Feb 23;12:641900. doi: 10.3389/fimmu.2021.641900 (PMC7959787; doi:10.3389/fimmu.2021.641900)
Supplement: Supplementary file 10 [file Data_Sheet_1.PDF]

Supplementary Material

1 SUPPLEMENTARY TABLES AND FIGURES

Supplementary Table 1

HLA class I genotypes of deceased patients and the control group.

Supplementary Table 2

GISAID identificators of used SARS-CoV-2 genomes.

Supplementary Table 3

Raw and processed HLA-peptide binding affinity matrices.

Supplementary Table 4

Statistical comparison of allele frequencies in deceased adults, elderly adults and the control group.

Supplementary Table 5

Percentage of the explained variance for HLA-A, HLA-B and HLA-C principal components.

Supplementary Table 6

Comparison of principal components and risk score between deceased adults, elderly adults and the control group.

Supplementary Table 7

Distribution of deceased adults, elderly adults and the control group in low, medium and high risk score groups.

Supplementary Figure 1.

Distribution of HLA-A, HLA-B and HLA-C alleles in risk score (RS) groups. Alleles with frequency over 5% in the cohort of deceased patients or in the control group are presented.

Supplementary Figure 2.

Age at death of homozygous and heterozygous deceased COVID-19 patients.
